# Awareness among resident doctors with regards to cardiac defibrillators

**DOI:** 10.4103/1658-354X.71574

**Published:** 2010

**Authors:** Rakesh Garg, Anju R. Bhalotra, Amit Pruthi, Poonam Bhadoria, Raktima Anand, Nishkarsh Gupta

**Affiliations:** *PGIMER and Dr. RML Hospital, New Delhi, India*; 1 *Department of Anaesthesiology and Intensive Care, Maulana Azad Medical College, New Delhi, India*

**Keywords:** *Audit*, *awareness*, *cardiac arrest*, *defibrillator*, *preuse check*

## Abstract

**Background and Aims:**

Electrical defibrillation is the most important therapy for patients in cardiac arrest. The audit was aimed to assess awareness among residents with respect to routine preuse checking of cardiac defibrillators.

**Materials and Methods:**

The audit was conducted at a multispeciality tertiary care referral and teaching center by means of a printed questionnaire from anaesthesiology residents. A database was prepared and responses were analyzed.

**Results:**

Eighty resident doctors participated in the audit. Most (97.8%) of the residents were sure of the presence of a defibrillator in the operation room (OR); 70% of postgraduates (PG)s were aware of the location of the defibrillator in the OR as compared to 83.7% of the senior resident (SRs). Also, 32.1% residents routinely check the availability of a defibrillator. The working condition of the defibrillator was checked by 21.7% of the residents; 25.3% ensured delivery of the set charge. Further, 8.2% of residents ensured availability of both adult and paediatric paddles. About 27.8% of residents ensured the availability of appropriate conducting gel and 53.8% residents were of the opinion that the responsibility of checking the functioning and maintenance of the defibrillators lies with themselves. Some 22% thought that both doctors and technical staff should share the responsibility, while 19.5% opined that it should be the responsibility of the technical staff.

**Conclusion:**

All medical equipment is to be tested prior to initial use and periodically thereafter. An extensive, recurring training program, and continued attention to the training of clinical personnel is required to ensure that they are proficient in the operation and testing of specific defibrillator models in their work area. We conclude that apart from awareness of the use of the equipment we are using, its preuse testing is must. All resident doctors should be aware of the presence and adequate functioning of the defibrillator in their ORs and this audit reinforces the need for training of all resident doctors.

## INTRODUCTION

Clinicians are responsible for continually improving the quality of care they provide and demonstrating this by setting and monitoring standards. Thus all healthcare professionals have a duty to audit their practice. Electrical defibrillation is the single most important therapy for the treatment of patients in cardiac arrest. This audit was aimed to assess awareness among residents working at a tertiary care referral center with respect to routine preuse checking of cardiac defibrillators. The outcome of the audit was aimed to suggest implementation of changes in the teaching protocols at individual, team, and service levels with the objective of improving health care delivery.

## MATERIALS AND METHODS

The audit was conducted at a multispeciality tertiary care referral and teaching center. This hospital has 25 operating rooms. The cases are referred from various parts of the country for complicated and specialty surgeries. The operation rooms (OR) are in located in OR complexes with different complexes for each speciality. The Department of Anesthesia has degree and diploma teaching courses in Anesthesiology. The department also appoints senior residents who work under the supervision and guidance of Anesthesiology consultants. The department appoints technical staff for assisting in the anesthesia related activities in the operation room and intensive care unit. They are also involved in the maintenance of the anesthesia related-equipments. The hospital has a biomedical engineering department involved in the maintenance of various equipments apart from maintenance service contracts from manufacturers.

The audit was performed by means of a printed questionnaire with questions pertaining to defibrillator peruse check distributed to the residents working in the Department of Anesthesiology. Residents included postgraduate students and senior residents. Postgraduate residents were medicos who were pursuing a three year training program in the specialty of Anaesthesiology and Intensive Care after completing their medical school (MBBS). Senior Residents were those medicos who held a degree in the specialty of Anaesthesiology and Intensive Care and were working as full time residents. A database was prepared and responses were analyzed.

The questionnaire given to residents is given below:

- Do you know whether a defibrillator is available in your OR?
  Yes / No/ Don’t know / Can’t say
- If yes, do you know exactly where is it kept?
  Yes / No/ Don’t know / Can’t say
- Do you check its availability daily in different ORs?
  Yes / No/ Don’t know / Can’t say
- Do you check whether it is in a proper working order daily?
  Yes / No/ Don’t know / Can’t say
- Do you check whether it is actually delivering the charge that it is set to deliver?
  Yes / No/ Don’t know / Can’t say
- Do you confirm the availability of both adult and paediatric paddles daily?
  Yes / No/ Don’t know / Can’t say
- Do you confirm the availability of conducting gel daily?
  Yes / No/ Don’t know / Can’t say
- Who do you think should be responsible for checking the functioning and maintenance of the defibrillators Doctors, OR Technical / Support Staff

## RESULTS AND OBSERVATIONS

Eighty resident doctors participated in the audit. Of these 34 were postgraduate students undergoing training in Anesthesiology (PG) and the remainders were Senior Residents (SR).

The questionnaire and pertinent responses given by the residents are given below:

- Do you know whether a defibrillator is available in your OR?
  For this question, 97.8% of the residents (97.7% of PGs and 98% of the SRs) were sure of the presence of a defibrillator in the OR. Of these, 100% of 3^rd^ year PGs and SRs were sure of the presence of a defibrillator in their ORs.
- If yes, do you know exactly where is it kept?
  Only 70% of PGs were aware of the exact location of the defibrillator in the OR as compared to 83.7% of the SRs. The awareness was better amongst senior PGs and SRs.
- Do you check its availability daily in different ORs? About 32.1% residents (20.3% PGs and 44% SR) routinely check the availability of a defibrillator daily in different ORs.
- Do you check whether it is in a proper working order daily?
  The working condition of the defibrillator was checked daily by only 21.7% of the residents (22.2% of PGs and 31% of SRs).
- Do you check whether it is actually delivering the charge that it is set to deliver?
  Only 25.3% ensured delivery of the set charge from the defibrillator. This checking was better in SRs (39%) as compared to PGs (11.7%).
- Do you confirm the availability of both adult and paediatric paddles daily?
  Only 8.2% of residents ensured availability of both adult and paediatric paddles daily.
- Do you confirm the availability of conducting gel daily?
  Only 27.8% of residents ensured the availability of appropriate conducting gel for the defibrillator. The response was better in SRs (40.7%) as compared to PGs (15%).
- Who do you think should be responsible for checking the functioning and maintenance of the defibrillators?
  Some 53.8% residents were of the opinion that the responsibility of checking the functioning and maintenance of the defibrillators lies with themselves and 22% thought that both doctors and technical staff should share the responsibility. Further, 19.5% were of the opinion that they should not be responsible for checking and maintenance; rather it should be the sole responsibility of the technical staff.

## DISCUSSION

The reported incidence of cardiac arrest in anesthetic cases in different studies has been quoted variably and ranges from 2.36 to 23.09 per 10,000.[1] The incidence of anesthesia-related cardiac arrest has been quoted variably in different surveys and studies and ranges from 0.5 to 2.10 per 10,000.[1] In a large survey of critical events in the OR, the incidence of cardiac arrest was 9.86 per 100,000 anesthesia cases.[2] In another survey, the cardiac arrest incidence was 34.6 per 10,000 anesthetic cases and author reported that 94.6% cardiac arrest occurred in the OR and rest in the post anesthesia care unit. Cardiac patients may develop life threatening arrhythmias in the perioperative period necessitating early defibrillation apart from correction of underlying causes.[3] The reported incidence of sudden cardiac arrest in a pregnant patient for cesarean section under epidural block during the operative period has been managed successfully due to early defibrillation.[4] So while managing such cases apart from other pertinent requirement, the defibrillator is a vital lifesaving device in the armamentarium of monitors and instruments.

Early defibrillation is critical to survival from sudden cardiac arrest mandating not only easy availability of a defibrillator at the site of arrest but also the doctors using it must be aware of its functioning and should have tested it thoroughly and sincerely. With every minute that passes between collapse and defibrillation the survival rates decreases.[5]

All medical equipment is to be tested prior to initial use and periodically thereafter. An extensive, recurring training programme and continued attention to the training of clinical personnel is required to ensure that they are proficient in the operation and testing of specific defibrillator models in their work area. The testing procedures used by clinical personnel should be developed jointly with biomedical engineering personnel and clinical staff.

The energy levels required for defibrillation have been recommended by the American Heart Association, necessitating that the defibrillator being used should deliver the set amount of energy. It is essential that the operator know whether the defibrillator is monophasic or biphasic, and hence, knows the appropriate charge to be delivered.

The defibrillator monitor shows the amount of energy set and energy delivered. This needs to be checked during routine preuse check of defibrillators. For checking, confirm that the defibrillator is connected to an AC power outlet. Turn the POWER switch on the front of the monitor to 200 joules. A baseline tract will appear on the scope along with ‘Defib On’ message. Press the ‘CHARGE’ button. When fully charged, depress the two paddle buttons together. ‘Test OK’ will appear on the scope and it will display 200 joules delivered.

All defibrillators including loaned or rented units must be tested prior to initial use and tested periodically as a part of scheduled maintenance. The frequency of company testing is determined locally taking into account the manufacturer’s recommendations. In no case should this testing be done less than biannually. Test criteria must ensure that they perform according to manufacturers specifications and also include an assessment of internal batteries. Batteries need replacement as least once every two years. Qualified biomedical engineering staff and / or contractors should conduct all scheduled maintenance tests and all results should be documented.

For routine daily checking of the defibrillator, one should physically inspect the defibrillator. Paddle integrity including cleanliness, functioning of the indicator lights such as battery charger, output energy, internal battery integrity and assessment and accessories of defibrillator must be checked daily.

A checklist must be developed in each institution to reduce equipment malfunction and operational error. Documentation of these operational tests must be ensured in an instrument logbook.

When a defibrillator fails, there are only two possible reasons: device failure and operator error. A recent working group of the United States Food and Drug Administration has reviewed data from both Medical Device Reporting System and from a five-state survey.[6] From these data, the working group concluded that the level of defibrillator failure is too high. In many cases, the source of the failure was not traced to the instrument, but to human error concerning care and maintenance of the device. Often inadequate user training and the failure of personnel to understand the need for daily equipment check led to failure during a cardiac emergency. The group also found that defibrillators and specific replaceable components (e.g., batteries) were kept in service beyond their useful life expectancy. In smaller hospitals and clinics, appropriate service engineers were either in short supply or totally absent leading to equipment deterioration.

In another survey, it was revealed that the resident doctors even had a problem in turning the defibrillator ‘on’ when required. Had they been checking the defibrillator, this problem would not have arisen, thus reemphasizing the need of routine check of medical equipments.[7]

Whether a defibrillator is monophasic or biphasic, manual or automatic, maintenance needs are similar. Preventive maintenance is done annually on a staggered schedule and according to the manufacturer’s guidelines. In addition to annual testing, unit staff members should do a check of the equipment at the start of every shift change. Following a brief checklist, the staff should do a test defibrillation, confirm that the equipment is charging correctly, and ensure that all the necessary supplies like electrodes and disposable patches are present. Making sure that the cover has no loose components; that all the cables of the device are without fissures, cuts, or broken wires, and that the exterior and the connector are cleaned and disinfected are also important to how well the equipment operates.[8]

The following recommendations are designed to assist user facilities and staff in minimizing these types of occurrences during both testing and patient defibrillation.[9]

- Perform a functional test of the defibrillator at least once a day or as per the facility’s or manufacturer’s protocol. Be sure to test the unit on battery power only. Be sure that all accessories such as ECG electrodes, lead wires, hands-free pads, cables, and paper are present. Check for expiration dates on disposable products such as electrodes and hands-free pads. Keep records of these tests. Report within your facility any failures including mechanical faults such as bent or broken connector pins and/or cables, and electrical malfunctions such as open circuits or failure to deliver selected energy level.
- Test the defibrillator system with an external load making sure to include the interface cable. Keep the hands-free interface cables plugged into the test load as a protective measure when the device is not in use if feasible. This measure can reduce the wear and tear from the frequent attaching and detaching of the connectors and the cables. The other ends of the cables can be connected to the defibrillator, if your facility’s protocol allows.
- Test the defibrillator system at least once every six months test to ensure that the system will reliably produce the maximum energy level that will be used on patients.
- Keep in mind that the interface cables (and other included wires) are subject to various mechanical bending and twisting during use. Functionally testing the cables may be helpful for early detection of connection issues which can cause intermittent opens/ shorts. Check the user manual for your particular make and model of defibrillator. Position the cables and connectors within easy reach of device users. This will prevent user over reaching and stretching during a code event that may lead to bending/breaking, incomplete mating and/or no connection of the pins.
- Train all device users to check the display screen and/ or to listen for an audible clicking sound. These are feedback mechanisms from defibrillators that confirm positive connections. Look for possible messages that may indicate a connection has or has not been established.
- Provide the most current instructions for use with each defibrillator model during daily testing and preventive maintenance procedures, including any manufacturer’s suggested checklists.
- Provide adequate staff training according to user facility’s policies and procedures.
- Provide the manufacturer’s technical support and emergency contact phone numbers near the defibrillator for easy contact if device does not operate properly.
- We conclude that apart from awareness of the use of the equipment we are using its preuse testing is must. Ventricular fibrillation (VF) is the commonest cardiac arrest rhythm and the only definitive treatment of VF is early defibrillation. It is mandatory that a preuse check is carried out as recommended. All resident doctors should be aware of the presence and adequate functioning of the defibrillator in their operating rooms and this audit reinforces the need for training of all resident doctors.
